# Gut Peptide GLP-1 and Its Analogue, Exendin-4, Decrease Alcohol Intake and Reward

**DOI:** 10.1371/journal.pone.0061965

**Published:** 2013-04-16

**Authors:** Rozita H. Shirazi, Suzanne L. Dickson, Karolina P. Skibicka

**Affiliations:** Department of Physiology, Institute of Neuroscience and Physiology, The Sahlgrenska Academy at the University of Gothenburg, Gothenburg, Sweden; University of Florence, Italy

## Abstract

Glucagon-like-peptide-1 (GLP-1) is a gut- and neuro-peptide with an important role in the regulation of food intake and glucose metabolism. Interestingly, GLP-1 receptors (GLP-1R) are expressed in key mesolimbic reward areas (including the ventral tegmental area, VTA), innervated by hindbrain GLP-1 neurons. Recently GLP-1 has emerged as a potential regulator of food reward behavior, an effect driven by the mesolimbic GLP-1Rs. Its role in other reward behaviors remains largely unexplored. Since a considerable overlap has been suggested for circuitry controlling reward behavior derived from food and alcohol we hypothesized that GLP-1 and GLP-1Rs could regulate alcohol intake and alcohol reward. We sought to determine whether GLP-1 or its clinically safe stable analogue, Exendin-4, reduce alcohol intake and reward. To determine the potential role of the endogenous GLP-1 in alcohol intake we evaluated whether GLP-1R antagonist, Exendin 9-39, can increase alcohol intake. Furthermore, we set out to evaluate whether VTA GLP-1R activation is sufficient to reduce alcohol intake. Male Wistar rats injected peripherally with GLP-1 or Exendin-4 reduced their alcohol intake in an intermittent access two bottle free choice drinking model. Importantly, a contribution of endogenously released GLP-1 is highlighted by our observation that blockade of GLP-1 receptors alone resulted in an increased alcohol intake. Furthermore, GLP-1 injection reduced alcohol reward in the alcohol conditioned place preference test in mice. To evaluate the neuroanatomical substrate linking GLP-1 with alcohol intake/reward, we selectively microinjected GLP-1 or Exendin 4 into the VTA. This direct stimulation of the VTA GLP-1 receptors potently reduced alcohol intake. Our findings implicate GLP-1R signaling as a novel modulator of alcohol intake and reward. We show for the first time that VTA GLP-1R stimulation leads to reduced alcohol intake. Considering that GLP-1 analogues are already approved for clinical use, this places the GLP system as an exciting new potential therapeutic target for alcohol use disorders.

## Introduction

Glucagon-like peptide-1 (GLP-1), released after meals and in association with gastric distension [1], is a potent anorexigenic peptide [2], [3], produced both in the periphery (pancreas, gut) and in discrete CNS sites. Within the brain, a major GLP-1 cell group arises from the nucleus tractus solitarius of the hindbrain [4] sending projections to many forebrain areas involved in feeding control [5], [6]. Therapeutic interest in the GLP-1 signaling system has focused largely on its incretin (insulin-releasing) properties, and has culminated in the discovery of a novel diabetes therapy, Exendin-4 (EX4), a long-acting GLP-1 analogue [7]. The observation that diabetic patients receiving EX4 therapy lose body weight [8] has intensified efforts to discover the neurobiological mechanisms/substrates downstream of GLP-1 signaling that mediate the weight loss effects of GLP-1. Recently, several studies have linked central GLP-1 receptor signaling to feeding control at the level of the mesolimbic reward system [9], [10], [11]. Given that common mesolimbic pathways confer reward from natural rewards (e.g. food) and drugs of abuse (e.g. alcohol)[12], we sought to determine whether the GLP-1 signaling system plays a role in alcohol intake and alcohol reward.

GLP-1 receptors are expressed in many brain areas relevant for reward-linked consummatory behaviors, including parts of the mesolimbic reward system such as the ventral tegmental area (VTA) [13]. Direct administration of EX4 into the VTA reduces food intake, food reward and food-motivated behavior [9]. Such a direct effect on the brain’s reward system might bring up the question of a potential role of GLP-1 to regulate rewarding consummatory behaviors that extend beyond feeding control, to those involved in reward more generally. A considerable body of evidence suggests a pivotal role for the VTA in the rewarding and reinforcing effects of alcohol [14]. Alcohol abuse is a widespread and debilitating disorder and there is therefore a great need for developing new, more effective pharmacotherapy. Hence the GLP-1 analogues already deemed clinically safe could be an attractive therapeutic option to reduce alcohol intake. However, the reduction of alcohol intake and the reduction of food intake do not always go hand in hand. Indeed, another clinically approved type 2 diabetes target, peroxisome proliferator-activated receptor-gamma, while effective at reducing alcohol intake surprisingly increases food intake [15]. In the case of GLP-1, however, there are indications that it might change food and alcohol intake in the same direction. Gastric bypass suppresses alcohol consumption in humans and this effect was potentially linked to elevated levels of GLP-1 [16].

In the present study, an over-arching hypothesis is that central GLP-1 signaling may provide a relevant therapeutic target, not only for disorders of feeding control, but also for substance use disorders such as alcohol use disorder. Specifically, we aim to show that enhanced GLP-1 signaling, by GLP-1 or EX4 administration, reduces alcohol intake in rats (in a free choice, limited access alcohol/water drinking paradigm) and reduces alcohol reward in mice (by suppressing the ability of alcohol to condition a place preference). Furthermore we identify the neuroanatomical substrate underlying the effect of the GLP-1R stimulation on alcohol consumption. Importantly, we also aim to uncover the contribution of the endogenous GLP-1 ligand to alcohol intake.

## Materials and Methods

### Animals

Male Wistar rats, weighing 250 g at the start of the experiments, or male NMRI mice weighing 22 g were supplied by Taconic (Bomholt, Denmark) or B&K Universal AB (Soletuna, Sweden). Lights were turned on at 6 am and off at 6 pm. All procedures were approved by the local Ethics Committee for Animal Experiments: Göteborgs djurförsöksetiska nämnd (GDN); permit number 199-11. Special care was taken to minimize the amount of animals used in this study and to minimize the amount of pain experienced by each animal in the studies described below.

### Drugs

GLP-1 (7-36), EX4 and Exendin-3(9-39) (EX9) were purchased from Tocris (Bristol, UK), dissolved in saline (vehicle for intraperitoneal (IP) administration) or artificial cerebrospinal fluid (aCSF; vehicle for all central injections) and stored as aliquots in −20°C. EX9 is a selective antagonist at the GLP-1R [17], [18]. Ethanol was dissolved in tap water to make a final 20% solution (v/v) for the voluntary drinking studies and dissolved in saline and injected IP for the conditioning study at a dose of 1.75 g/kg (in NMRI mice). Doses for IP administration of GLP-1, EX4 and EX9 were based on previous studies [19] and shown to have a mild anorexic or orexigenic (EX9) effect in low-fat fed rats, though were without effect on high-fat fed rats. Intra-VTA doses were shown previously to reduce food-reward behavior [9].

### Intermittent-Access 20% Ethanol 2-Bottle-Choice Drinking Model

This model was adapted from [20] and [21]. Rats were given access to a 20% ethanol solution for three 24 h sessions per week, separated by a minimum of 24 h and a maximum of 48 h (weekends) of no access to ethanol (where the ethanol solution bottle was replaced by a second water bottle). To control for side preferences the placement of the ethanol bottle was alternated each ethanol session. Rats were weighed six days per week to calculate ethanol intake per kg of body weight. Drug testing began 4 weeks (or total of 12 ethanol sessions) from the first ethanol exposure. This period creates a stable ethanol intake in Wistar rats, comparable with that of the alcohol-preferring rats [21]. Rats had unlimited access to chow and water at all times and intake of both was measured together with the ethanol measurements. For each experiment the following conditions were used: **I)** For IP GLP-1 application: vehicle or GLP-1 0.1 mg/kg injections at 1 ml/kg; **II)** For IP EX4 application: vehicle, EX4 0.3 mg/kg or EX4 1.0 mg/kg at 1 ml/kg; III**)** For IP EX9 application: vehicle or EX9 0.1 mg/kg. Injections were always completed 30 min before ethanol exposure. With the exception of GLP-1, all comparisons of drug to vehicle treatment were made between-subject. In order to compare baseline-vehicle drinking to that of GLP-1, the same rats received counterbalanced conditions on two separate drinking sessions.

### Conditioned place preference (CPP)

The CPP test was performed in NMRI mice. We decided to utilize a mouse CPP model in accordance with the past literature and in house preliminary testing indicating that mice, unlike rats, show a reliable and reproducible preference for the ethanol paired compartment. The CPP apparatus (Med Associates, MED-CPP2-RS, ST Albans, VT, USA) comprised two chambers, each with distinct visual and textile cues. The procedure consisted of preconditioning on day 1 (in which mice were IP-injected with vehicle and initial place preference was determined during 30 min), conditioning on days 2–8 (in which the least preferred compartment was paired with alcohol injection), and postconditioning on day 9 (in which the preference for the alcohol paired compartment was assessed during 30 min). Before the test session (on the postconditioning day) NMRI mice received an IP injection of GLP-1 (0.02 mg in 0.2 ml) or vehicle (0.2 ml of saline).

### Brain cannulation

For behavioral studies targeting the CNS, a VTA guide cannula was positioned and attached to the skull with dental acrylic and jeweler's screws and closed with an obturator under ketamine/xylazine anesthesia, as described previously [22]. Briefly the guide cannula (26 gauge; Plastics One, Roanoke, VA) coordinates used were: ±0.75 mm from the midline, 5.7 mm posterior to bregma, and 6.5 mm ventral from the surface of the skull, with injector aimed 8.5 mm ventral to skull. At the end of the study, rats were deeply anesthetized with isoflurane and euthanized by cervical dislocation. Subsequently, the brain was removed for the VTA placement confirmation. The VTA placement was verified post mortem by microinjection of India ink at the same volume (0.5 μl) used throughout the study. Only rats with the correct placement were included in the analysis. One week post-surgery, rats were returned to the intermittent drinking schedule. Post-surgery drinking baseline was reduced compared to pre-surgery intake. The following experiments were conducted utilizing the VTA guide cannula: **I)** the effect of GLP-1 on ethanol intake in which rats received unilateral microinjection of either vehicle (n = 11) or 1.0 µg GLP-1 (n = 7) and **II)** the effect of EX4 on ethanol intake in which rats received unilateral microinjection of either vehicle (n = 9) or 0.1 µg EX4 (n = 9).

### Statistics

Statistical analysis utilized Graph Pad Prism (San Diego, CA) software. Data are reported as mean ±SEM. Effects were evaluated using a Student’s t test or within- or between-subjects one or two-way ANOVA, as appropriate. Post hoc comparisons were made with Tukey’s test. p values of <0.05 were considered significant.

## Results

### Peripheral injection of GLP-1 or GLP-1 analogue reduces alcohol intake

**Figure 1:**
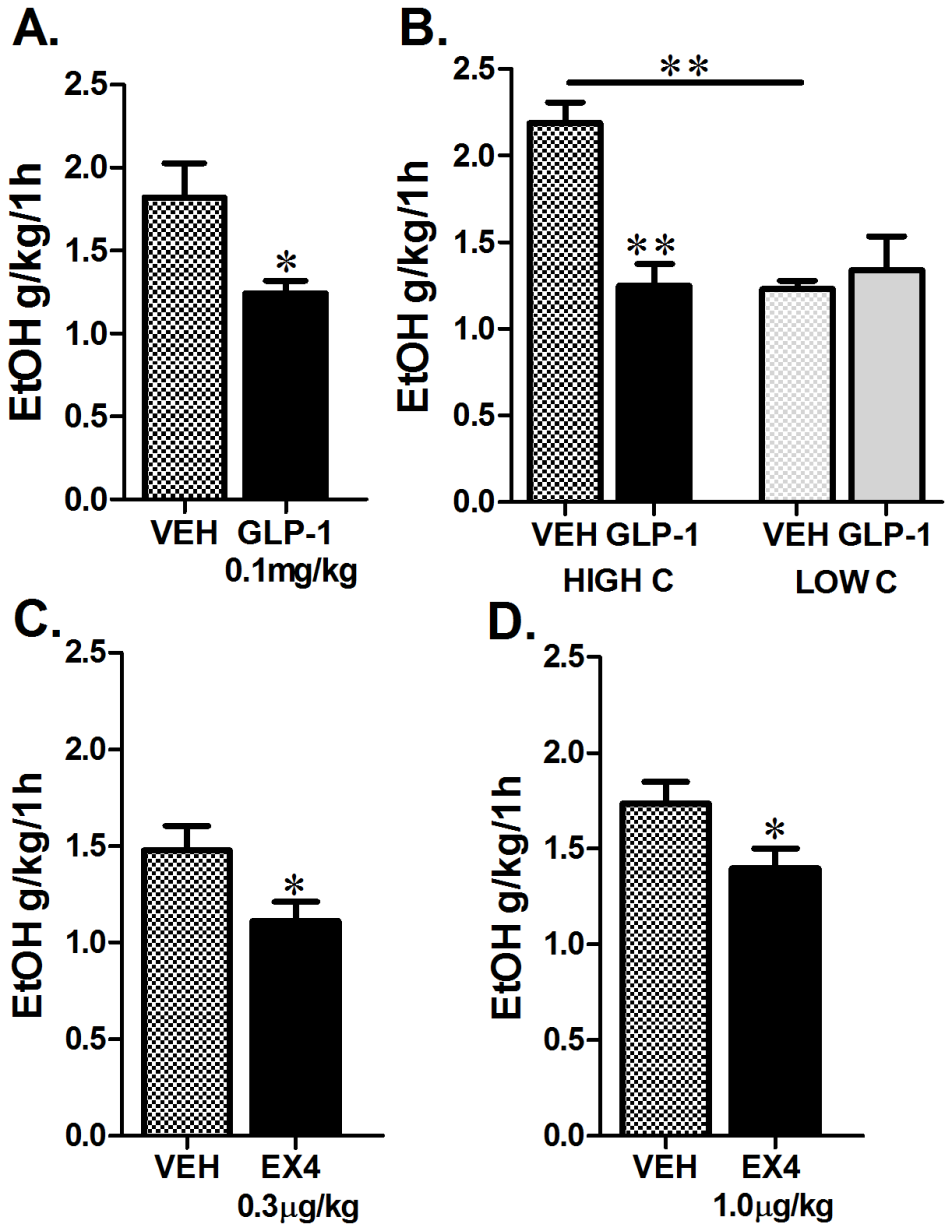
Peripheral administration of GLP-1 or EX4 reduces voluntary alcohol intake. In an intermittent-access 20% ethanol drinking paradigm, Wistar rats peripherally injected with GLP-1 (0.1 mg/kg) drank less alcohol than those injected with vehicle at 1 h (n = 12 per treatment group (A)). The reduced alcohol drinking response was primarily exhibited by high alcohol consuming rats (HIGH C, top 30% consumers) and was not detected in low alcohol consuming rats (LOW C; bottom 30%) (B). Rats that received an IP injection of EX4 at a dose of either 0.3 µg (C) or 1.0 µg/kg (D) reduced their 20% ethanol intake at 1 h after alcohol exposure n = 13−25. All values represent mean ± SEM. VEH, vehicle for GLP-1 (glucagon-like-peptide-1); EtOH, ethanol. *p<0.05, **p<0.01.

Relative to vehicle-injected rats, those that received IP GLP-1 reduced their alcohol consumption by nearly 30% over the first hour of ethanol exposure (n = 12 per treatment group, students t-test, p<0.05, Figure 1A). When the vehicle baseline drinking was used to separate the rats into high and low drinking groups (upper and lower thirds of original n = 12 based on 1 h ethanol intake), an interesting interaction emerged between this baseline alcohol consumption and the efficacy of GLP-1 to reduce alcohol intake (two-way ANOVA baseline drinking vs. drug treatment; interaction F_(1,12)_ =  32.5, p<0.005). A potent effect of GLP-1 emerged but only in the high drinking group (Tukey’s test, p<0.01; Figure 1B). Notably the same high and low alcohol consumers did not differ in their vehicle baseline water (high alcohol drinking group: 2.2±0.3 ml vs. 3.4±1.0 ml for vehicle vs. GLP-1 respectively, p = ns; low alcohol drinking group: 5.6±1.3 ml vs. 7.6±2.8 ml for vehicle vs. GLP-1 respectively, p = ns) or chow consumption (high alcohol drinking group: 4.6±0.8 g vs. 3.4±0.4 g for vehicle vs. GLP-1 respectively, p = ns; low alcohol drinking group: 4.4±0.6 g vs. 4.3±0.9 ml for vehicle vs. GLP-1 respectively, p = ns) and GLP-1 did not affect water or chow consumption in these subgroups. Importantly, as shown in Figure 1A, this division was not a prerequisite to uncover the effect of GLP-1 on alcohol drinking, since even when all rats are included there is still a clear and significant effect of GLP-1 on drinking. This division was applied only for this first experiment (data presented in Figure 1B) and thus all remaining data described below includes all consumers and what follows the natural variability in drinking without any pre-selection.

Peripheral injection of EX4, significantly reduced 1 h alcohol consumption at both 0.3 and 1.0 µg/kg doses (students t-test, p<0.05 for both the 0.3 and 1.0 µg/kg doses; Figure 1 C, D). The selectivity of the effect towards alcohol and not general liquid intake was supported by our data showing that 0.3 µg dose of EX4, while effective at reducing alcohol intake, did not significantly alter water or food intake (data not shown). The higher 1.0 µg dose did not alter 1 h food intake; it did, however, reduce 1 h water intake (4.4±0.5 ml vs. 2.5±0.4 ml for vehicle vs. EX4 respectively, p<0.005).

### Peripheral injection of GLP-1 reduces reward derived from alcohol

**Figure 2:**
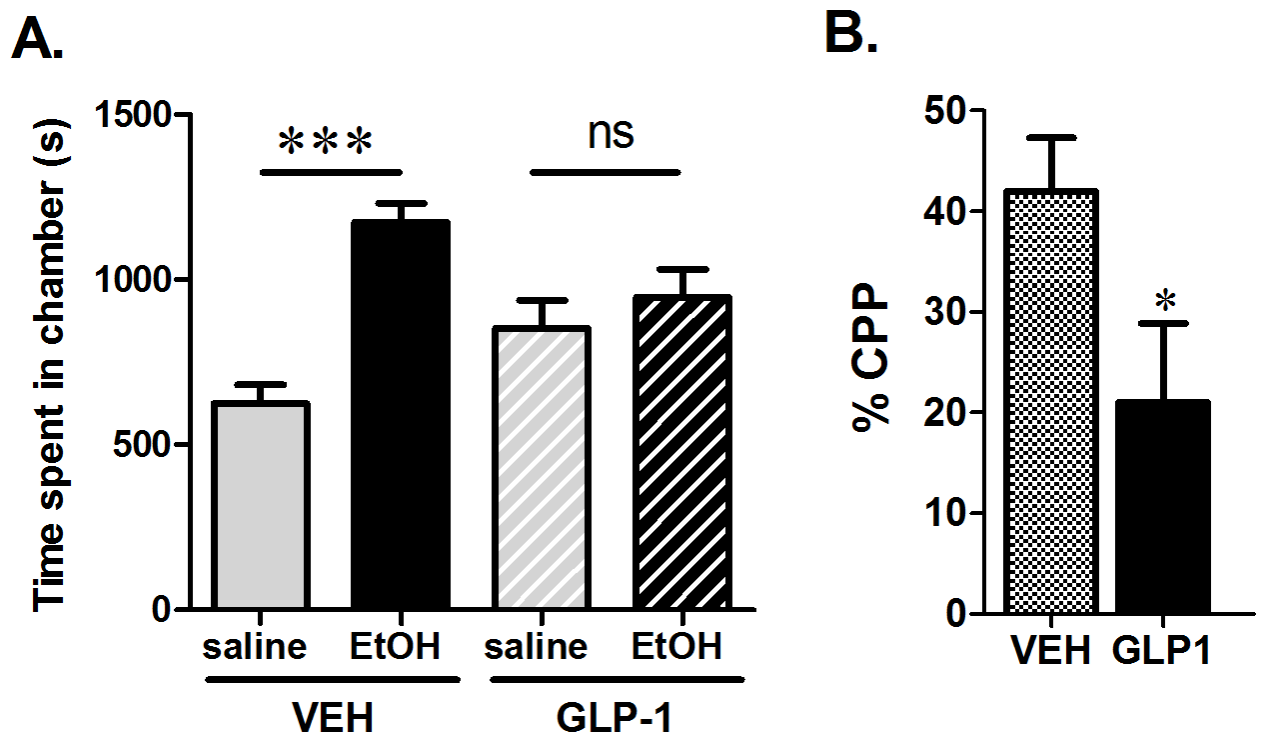
Peripheral administration of GLP-1 reduces alcohol reward. Mice treated with vehicle on the testing day spent significantly more time in the compartment previously (during the conditioning sessions) paired with alcohol as compared to the compartment paired with saline. In contrast, mice treated with 0.02 mg of GLP-1 spent an equal amount of time in both the saline- and alcohol-paired compartments (A). Alcohol induced a significant preference for the compartment it was paired with over the compartment paired with saline during the conditioning sessions in NMRI mice injected with vehicle (n = 48) but not those treated with GLP-1 (n = 31) (B). %CPP was determined with the following formula ((test-pretest)/(total time- pretest))×100 to indicate the % preference above a neutral response (i.e. equal preference for each compartment). All values represent mean ± SEM. *p<0.05, **p<0.01.

In the CPP test, NMRI mice (n = 31−48) showed a preference for the compartment previously paired with daily alcohol injections over one week, reflected by an increased time spent there (Figure 2A, one-way ANOVA, F _(3,154)_ = 13.5, p<0.0001). Post hoc Tukey test revealed a significant difference (p<0.001) between saline vs. alcohol conditioned compartment time in mice treated with vehicle on the testing day. In contrast the mice treated with GLP-1 on the testing day did not differ in the amount of time spent in saline vs. alcohol-paired compartment. Injection of GLP-1 at the testing phase significantly reduced this preference (Figure 2B, % CPP preference of vehicle vs. GLP-1 t-test: p<0.05), suggesting that GLP-1 can interfere with rewarding aspects of alcohol.

### Blockade of GLP-Rs increases alcohol consumption

**Figure 3:**
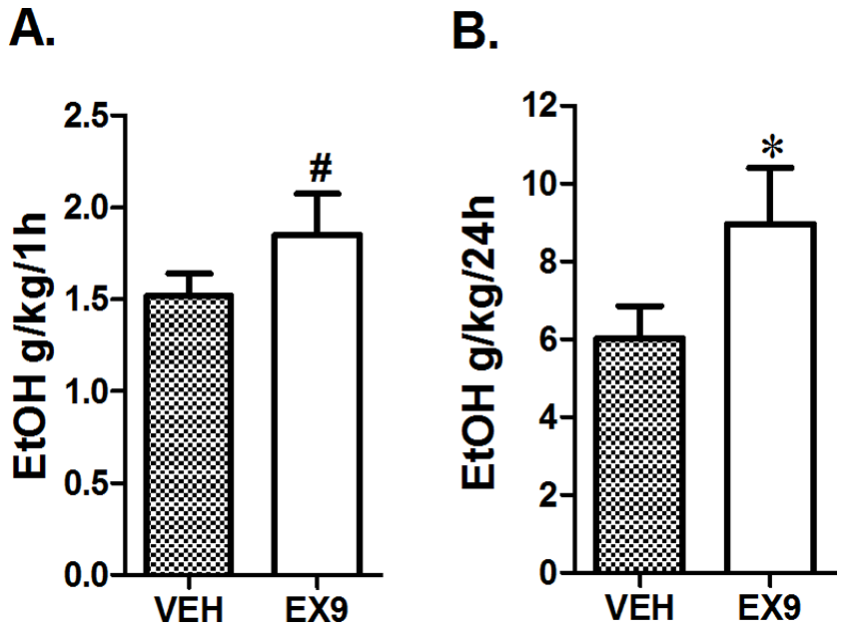
Blockade of GLP-1Rs increases voluntary alcohol intake. Rats (n = 12−13) peripherally injected with 0.1 mg/kg EX9, a selective GLP-1R antagonist, displayed a tendency for increased consumption of 20% ethanol during the first hour of alcohol exposure (A) that reached significance at the 24 h measurement time-point (B). All values represent mean ± SEM. ^#^p<0.1, *p<0.05.

Peripheral injection of the selective antagonist of the GLP-1R, EX9, resulted in a trend to increase alcohol consumption at 1 h (t-test, p = 0.09, Figure 3A), which became significant at the 24 h measurement time point (n = 12−13, t-test, p<0.05, Figure 3B). Neither food nor water intake was altered by the treatment (data not shown).

### VTA selective GLP-1 and EX4 application

**Figure 4:**
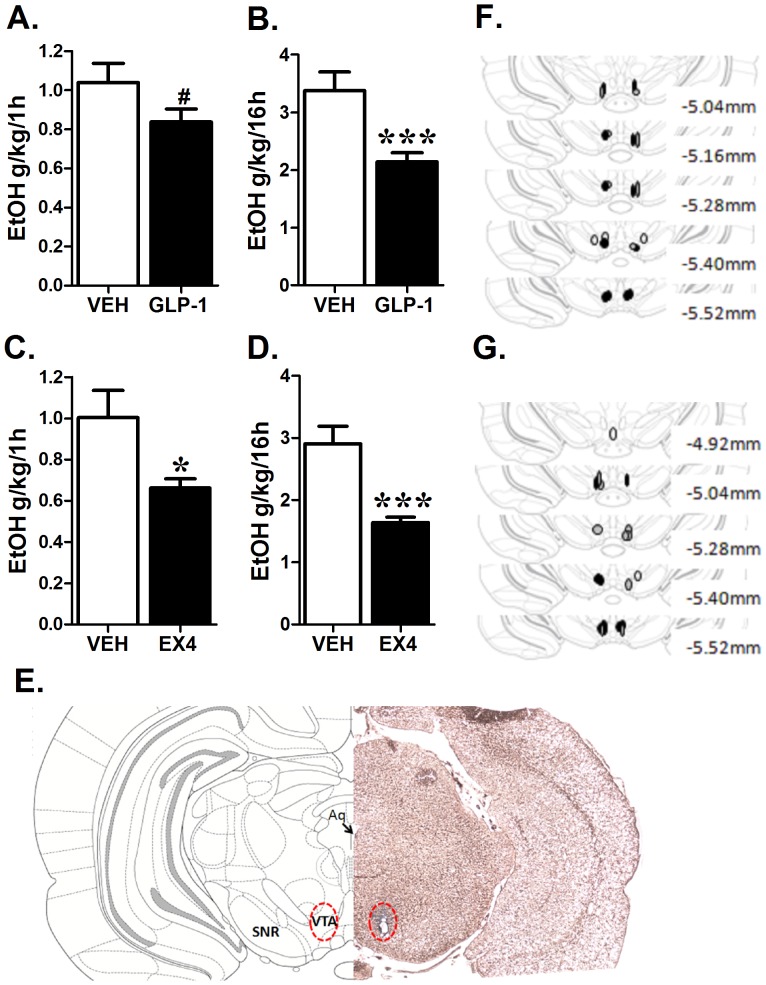
Identification of the mesolimbic VTA as the neuroanatomical substrate for GLP-1R-linked effects on alcohol consumption. VTA-selective unilateral microinjections of GLP-1 (vehicle n = 11; GLP-1 1 µg n = 7, A−B) and EX4 (vehicle n = 9; EX4 0.1 µg n = 9, C−D) reduced 20% ethanol consumption during a 16 h drinking session. A diagram based on Paxinos and Watson at the level of bregma −5.40 mm shows a representative VTA injection site (E). Additionally, schematics illustrate the injection site for each rat from the GLP-1 (F) and the EX4 (G) study. Black circles represent vehicle-injected rats, grey drug-injected and white missed placement. All values represent mean ± SEM. Aq; aqueduct, SNR; *substantia nigra pars reticulata*. ^#^p<0.1,*p<0.05, ***p<0.005.

Microinjection of GLP-1 selectively to the mesolimbic VTA reduced alcohol consumption (t-test, p<0.1 and p<0.005 for 1 and 16 h respectively; Figure 4 A−B). The same treatment did not alter overnight water intake (28.7±2.6, 24.9±3.0 ml of water drank for vehicle and GLP-1 respectively, ns) or food intake (22.5±0.7, 20.6±2.9 g of chow eaten, ns). A similar effect was obtained with VTA-directed microinjection of EX4 (Figure 4 C−D). EX4 reduced both 1 h and 16 h ethanol intake (students t-test, p<0.05 and p<0.005 for 1 and 16h respectively). Unlike GLP-1, VTA directed EX4 significantly reduced water intake (29.0±4.7, 13.8±1.5 ml of water drank for vehicle and EX respectively; t-test, p<0.05). The vehicle baseline alcohol drinking for intra-VTA applied vehicle (acsf) was lower than that measured with IP vehicle injection (saline); this parallels what we observed previously for food reward behavior [23] and could potentially result from an overall impact of the central injection on behavior. Intra-VTA EX4 did not change the 1 h but reduced the 16 h food intake (1 h: 4.5±0.5 vs. 4.6±0.2 g, ns; 16 h: 22.8±0.8 g vs. 14.3±0.9 g of chow eaten for vehicle vs. EX4, t-test, p<0.005). Two animals received GLP-1 microinjections just outside of the VTA (dorsally and laterally); their 1 h and 16 h ethanol intake were not significantly altered compared to vehicle controls.

## Discussion

The present studies reveal an important role for GLP-1 in alcohol consumption and reward. Peripherally applied GLP-1 reduced consumption of a 20% ethanol solution and suppressed alcohol reward in the CPP test. EX4, a stable analogue of GLP-1 approved for clinical use in diabetic patients, was similarly able to reduce voluntary alcohol drinking. Furthermore our novel findings, that VTA stimulation of GLP-1Rs is sufficient to reduce alcohol drinking, point to this mesolimbic structure as the neuroanatomical substrate underlying the suppressive effect of GLP-1 on alcohol drinking. These results suggest that GLP-1R stimulation is sufficient to drive a reduction in ethanol-oriented behavior but do not attend to the issue of the involvement of endogenous GLP-1 in the regulation of alcohol drinking. However, a role for endogenously released GLP-1 in alcohol consumption is suggested by our results indicating that blockade of GLP-1Rs increases alcohol consumption.

The current results, together with recent findings indicating an important role of GLP-1 agonists in food motivation/reward [9], amphetamine- [24], and cocaine- [25] induced behavior, highlight a potentially comprehensive role for GLP-1 in reward behavior control. The reason for this broad-spectrum effect of GLP-1 agonists might lie in the convergence of the neural substrate for all reward behaviors on the mesolimbic and/or nigrostriatal pathways. Notably the neuroanatomical distribution of the GLP-1R supports this hypothesis, as GLP-1R has been located in both key mesolimbic (VTA and NAc) and nigrostriatal structures in addition to its well-characterized hypothalamic distribution [13]. This resonates with the emerging concept that appetite-regulating peptides such as ghrelin, leptin, insulin, NPY and orexin, directly target the brain’s reward system where they serve to regulate “addictive” consummatory behaviors that extend beyond feeding control, to those involved in reward more generally [26], [27]. A key finding of this paper points to the mesolimbic VTA as an important neural substrate for the effects of GLP-1 on alcohol drinking.

The neurotransmitters downstream of GLP-1R activation leading to changes in reward behavior are not yet known. The VTA dopamine neurons provide a common mediator for drug and food reward behaviors and thus, are a likely downstream candidate. Notably preliminary data suggest that GLP-1R might be expressed on dopaminergic neurons in the VTA, making dopamine a possible downstream target [28]. That EX4 decreases amphetamine-induced hyperactivity and reward derived from cocaine, behaviors inherently linked to the mesolimbic dopamine, further strengthens the idea of a potential connection between the GLP-1 system and dopamine [24], [25]. Interestingly, it is not only the tegmental dopamine neurons that might receive input from the GLP-1. Emerging literature points to the substantia nigra dopamine neurons as another potential target for GLP-1. In fact this discovery has already been exploited in preclinical models of Parkinson’s disease and ongoing clinical trials [29], [30]. Notably GLP-1Rs in the VTA do not have to be directly present on the dopamine neurons to influence dopaminergic function. They could be located on GABAergic or glutamatergic neurons in the VTA thereby regulating dopamine transmission indirectly.

While our results clearly point to the VTA GLP-1R population as sufficient to alter alcohol intake, they do not eliminate the possibility that other brain GLP-1R-expressing populations might play a role in alcohol regulation in addition to those in the VTA when the agonist is applied systemically. In fact GLP-1Rs in the NAc [13] represent another interesting target with respect to reward behavior as their neuroanatomical location in the projection target of the VTA dopaminergic neurons might allow them to pre-synaptically regulate dopamine release or post-synaptically act on dopamine target neurons. Furthermore many of the hypothalamic (e.g. lateral hypothalamus) and brainstem (e.g. nucleus tractus solitarius) nuclei that densely express GLP-1Rs [13] are either directly or indirectly linked to the mesolimbic VTA allowing them to potentially influence the mesolimbic function.

Our results showing a role for GLP-1 in alcohol consumption are not the first to connect appetite-regulatory anorexigenic peptides to alcohol consumption. Previous studies indicate that, for example, melanocortins and cholecystokinin can also reduce alcohol intake e.g. [31], [32]. Notably both peptides have previously been shown to interact with GLP-1. Interestingly, as already mentioned, there is also precedence for approved anti-diabetic treatments (thiazolidinediones, pioglitazone and rosiglitazone) to reduce alcohol intake and reward [15]. Collectively, these data highlight the overlap and the neural crosstalk between pathways controlling food intake, blood glucose and alcohol drinking.

The potential link between GLP-1 and alcohol intake initially surfaced from the field of bariatric (weight loss) surgery. Surgical procedures such as Roux-en-Y gastric bypass have been reported to suppress consummatory behaviors, even for alcohol, effects potentially associated with reduced circulating levels of ghrelin and elevated levels of GLP-1 (compared to non-operated littermates) [16]. Interestingly, in this gastric bypass study while EX4 reduced alcohol intake in control rats, it was ineffective at reducing ethanol drinking in RYGB rats, i.e. rats that already have reduced alcohol consumption as a result of the surgery [16]. This finding is perhaps mirrored in our current results indicating that GLP-1 stimulation is most effective in those rats that consume the highest amount of alcohol and ineffective in their low drinking littermates. This selective potent effect in highest alcohol consumers might, of course, be of clinical advantage.

Alcohol, in addition to its direct reinforcing effect, can also provide a source of fluid and calories. For that reason a potential action of GLP-1 to generally reduce caloric intake rather than specifically reduce alcohol intake should be considered. In our rat studies, however, the collective data clearly point to a role of GLP-1 in ethanol consumption that is dissociable from its effects on fluid and caloric consumption. Water and food intake, in contrast to alcohol intake, were not altered by any of the GLP-1 treatments (IP or VTA) at any time point tested making an overall effect on satiety an unlikely explanation for the alcohol intake suppressive effects of GLP-1. Furthermore, alcohol represented only a small portion of calories consumed: at the 1 h measurement point calories consumed from alcohol were 7 to 14 and 40 to 50 kcal/kg per rat for alcohol and chow respectively. Note also the lack of effect of the GLP-1R antagonist, EX9, on food or water intake when a clear effect on alcohol consumption was present. This dissociation of effect was also noted for the lower dose of EX4 applied peripherally, though the higher dose and also intra-VTA EX4 reduced water intake. This may seem an unexpected divergence of effects between EX4 and GLP-1. However, even though both clearly target the same receptor, there are significant differences in potency and stability between them. There is emerging evidence that while EX4 is highly specific to the GLP-1R, the molecular and behavioral consequences of its receptor activation might be slightly different to those determined for GLP-1 [33], [34]. These differential effects of the two GLP-1R ligands might have contributed to the divergence of effect on alcohol vs. water intake observed here. Collectively, our data point to the possibility of reducing alcohol intake via GLP-1R stimulation without producing a simultaneous reduction in food and water intake. Moreover, GLP-1 reduced the ability of alcohol to condition a place preference in mice, an effect that reflects the reward of prior alcohol exposure. Note that no calories in any form (food or alcohol) are available during this test.

Interestingly, current results might also suggest higher sensitivity of alcohol intake behavior compared to water/food intake behavior to EX4, as a higher dose is needed to affect the latter. The overall lack of effect of GLP-1 or EX4 on food intake might be surprising; the doses used were low, however, and were previously shown to be ineffective in changing food intake in high-fat fed rats [19]. Indeed, it is possible that several weeks of alcohol drinking may have contributed to a reduced efficacy of GLP-1R agonists on food intake, since as mentioned above alcohol can provide an additional source of calories. Whether chronic alcohol intake, or the additional calories ingested by chronic alcohol intake, induces a reduced sensitivity to the anorexic effects of GLP-1R stimulation similarly to what has been noted in rats after a period of high-fat consumption [19] is an intriguing idea that may warrant further investigation.

An alternative explanation for the suppressive effects of GLP-1 on alcohol intake could involve induction of nausea or aversion, effects that have previously been linked to GLP-1 analogues [35], [36]. This seems unlikely as multiple publications indicate that while the activation of GLP-1Rs in the hindbrain and amygdala might result in nausea, this effect does not extend to the mesolimbic GLP-1R population [9], [10], [11]. Additionally, the peripheral doses of EX4 used here were not sufficiently high to produce taste or place aversion [24], [37].

Collectively our findings reveal that GLP-1 and its mesolimbic receptors can modulate alcohol intake and alcohol reward. Current results also implicate for the first time the endogenous GLP-1 in regulation of alcohol intake. These preclinical studies point to the possibility that GLP-1 analogues could be considered for the treatment of alcohol use disorder. Considering that GLP-1 analogues are not only already approved for clinical use, but may also offer additional benefits in the form of neuroprotection [30], [38], [39], they are certainly an attractive therapeutic target warranting further evaluation for their potential use in the treatment of alcohol use disorders.
